# European Society for Organ Transplantation (ESOT) Consensus Statement on Outcome Measures in Liver Transplantation According to Value-Based Health Care

**DOI:** 10.3389/ti.2023.12190

**Published:** 2024-01-25

**Authors:** Marco Carbone, James Neuberger, Ian Rowe, Wojciech G. Polak, Anna Forsberg, Constantino Fondevila, Lorenzo Mantovani, Alessandra Nardi, Agostino Colli, Karen Rockell, Liz Schick, Laura Cristoferi, Gabriel C. Oniscu, Mario Strazzabosco, Umberto Cillo

**Affiliations:** ^1^ Department of Medicine and Surgery, University of Milano-Bicocca, Milan, Italy; ^2^ Liver Unit, ASST Grande Ospedale Metropolitano (GOM) Niguarda, Milan, Italy; ^3^ Liver Unit, Queen Elizabeth Hospital, Birmingham, United Kingdom; ^4^ School of Medicine, University of Leeds, Leeds, United Kingdom; ^5^ Erasmus MC Transplant Institute, Erasmus University Medical Center Rotterdam, Rotterdam, Netherlands; ^6^ Institute of Health Sciences, Lund University, Lund, Sweden; ^7^ General and Digestive Surgery Service, Hospital Universitario La Paz, Madrid, Spain; ^8^ Center for Study and Research on Public Health, University of Milan-Bicocca, Milan, Italy; ^9^ Department of Mathematics, University of Rome Tor Vergata, Rome, Italy; ^10^ Istituto di Ricovero e Cura a Carattere Scientifico, Ca’ Granda Foundation Maggiore Policlinico Hospital, Milan, Italy; ^11^ Independent Researcher, Bristol, United Kingdom; ^12^ World Transplant Games Federation, Winchester, United Kingdom; ^13^ Department of Medicine and Surgery, University of Milano Bicocca, Monza, Italy; ^14^ Division of Transplantation Surgery, Department of Clinical Science, Intervention and Technology, Karolinska Institute, Stockholm, Sweden; ^15^ Department of Internal Medicine, School of Medicine, Yale University, New Haven, CT, United States; ^16^ Department of Surgical, Oncological and Gastroenterological Sciences, School of Medicine and Surgery, University of Padua, Padua, Italy

**Keywords:** liver transplantation, value-based health care, PROM, wait-list, outcomes ATS

## Abstract

Liver transplantation is a highly complex, life-saving, treatment for many patients with advanced liver disease. Liver transplantation requires multidisciplinary teams, system-wide adaptations and significant investment, as well as being an expensive treatment. Several metrics have been proposed to monitor processes and outcomes, however these lack patient focus and do not capture all aspects of the process. Most of the reported outcomes do not capture those outcomes that matter to the patients. Adopting the principles of Value-Based Health Care (VBHC), may provide an opportunity to develop those metrics that matter to patients. In this article, we present a Consensus Statement on Outcome Measures in Liver Transplantation following the principles of VBHC, developed by a dedicated panel of experts under the auspices of the European Society of Organ Transplantation (ESOT) Guidelines’ Taskforce. The overarching goal is to provide a framework to facilitate the development of outcome measures as an initial step to apply the VMC paradigm to liver transplantation.

## Introduction

Liver Transplantation (LT) is a complex procedure surgically, medically, and ethically, and by necessity, a highly regulated field. It is expensive in terms of costs and resources but improves the quality and length of life of patients with end-stage liver disease [1]. LT is not a single care episode, but rather a life-long process that includes several sequential steps, from referral, to evaluation, list management, maintenance of fitness for transplantation, surgery, and life-long follow-up, which includes maintenance of patient and graft health, management of immunosuppression and, sometimes, hospitalization and additional surgeries [2]. Each of these steps requires adaptation of the recipient’s everyday life and strongly impacts their quality of life and expectations.

LT is an ideal field for application of the Value-Based Health Care (VBHC) approach, but to our knowledge, this has not yet been attempted [3, 4]. A recent systematic review of quality metrics in liver transplantation identified 317 quality metrics condensed into 114 indicators. Measures were focused primarily on safety and effectiveness, but very few addressed equity and patient centeredness [5]. Furthermore, these measures were mostly process indicators. Process indicators are intended as a help to improve outcomes, but do not measure whether the desired outcome is reached. Furthermore, most studies report outcomes in terms of patient and graft survival censored at relatively short intervals after transplantation [6–8] and miss important steps of the transplant journey such as quality of life before and after transplant, death awaiting liver transplant or complications late after transplantation [9–11].

In the early years of liver transplantation, outcomes focused on short term outcomes (such as 1 year post-transplant patient survival, incidence of rejection or in-patient stay). As outcomes have improved, additional metrics have increasingly been introduced to measure the quality of liver transplantation. However, it is worth noting that, in many cases, regulatory bodies still emphasize patient and graft survival [11, 12]. This represents a simplistic, flattened, and one-dimensional description of the highly complex process of liver transplantation.

Therefore, there is a critical need to identify metrics that offer not only those that meet the patient’s needs and wishes but also provide a more comprehensive measurement of the quality of the process.

Developing a culture of quality improvement means setting goals, measuring processes and outcomes, developing action plans where indicated, and assessing the impact of any change [13, 14]. The final aim should not be to equal or improve established benchmarks, but rather to develop a system that continually redefines the benchmarks to yield optimal patient care, and increased patient-level value. In this context, a paradigm change in this direction is needed to better realign clinical endpoints to patients’ needs and expectations.

Such a change in paradigm can leverage on the model of VBHC. VBHC is about delivering health outcomes that truly matter to patients. Value in healthcare is defined as patient-level outcomes divided by the cost to achieve those outcomes [3]. In essence, it means delivering the best possible outcomes at the right cost and orienting the competition towards increasing the value for the patients. This definition was introduced by Michael Porter and Elizabeth Teisberg in 2006 in a publication that originated the entire field of Value-Based Health Care [13, 15].

The VBHC proposition has been applied to several conditions, hospitals and units and healthcare systems [16–20]. However, a lack of clarity regarding the definition of value has led to divergent approaches. Value, according to Porter and Teisberg, is not synonymous with lower costs or higher revenues, and is more than cost-effectiveness. The numerator of the value ratio are condition-specific outcomes that are important to patients with that condition. The denominator is the total spending for the full cycle of care. Most healthcare quality research focused on process measures, while outcomes vary dramatically and are mostly left unmeasured. VBHC differs from simple quality measurement or improvement in that it assesses the quality of the whole system from the patient’s perspective [16].

The ESOT Guidelines Taskforce agreed that survival alone is a limited measure for outcome after LT and considered the need for a re-evaluation of LT outcome endpoints as a clinical and scientific priority for the Society. In particular, the Taskforce acknowledged that there is a need to look for multiple, complementary patient-centered metrics that capture the whole transplant process from a VBHC perspective; such metrics include waitlist outcomes, post-transplant complications, survival and measures of health-related quality of life. These metrics would enhance patient-level decision-making, provide evidence on LT effectiveness, benefits and complications and allow comparisons with alternative therapeutic interventions. In time, such metrics will enable benchmarking and comparison across centers and countries to assess differences and benefits of their respective processes.

Three sets of data are needed to implement a VBHC approach: clinical outcome indicators, patient-reported outcomes, and costs (or resource utilization). As a first step, the Taskforce aimed to reach a consensus on a set of clinical outcomes indicators that assess the whole process of LT from a VBHC perspective. Further work will need to focus on developing a set of patient reported outcomes and costing assessment methods allowing comparison among different healthcare systems and jurisdictions.

## Methods

The consensus development process was organized by a dedicated Guidelines Taskforce within ESOT and its sections ELITA, EKITA, EPITA, ECTTA, ETHAP, Education Committee, YPT, Transplant International editorial board members and patient representatives (Table 1).

**TABLE 1:** Topics and questions formulated by the panel, and authors of the literature review for each topic.

Topic	Questions		Author/s
Waiting list management	In a setting with optimal potential candidate, referral and listing process, which is the best measure to evaluate the quality of waiting list management in a VBHC perspective?		Strazzabosco M
Quality of life after LT	Which is the best tool to measure health-related quality of life, when assessing benefit of liver transplantation?		Neuberger JM
Patient reported outcome and experience measures	What are the unmet needs in defining the critical PROMs and PREMs to be included in liver transplant “core” evaluation and clinical trial design?		Rowe I
Timeframe for outcomes comparison	What is the most appropriate timeframe to describe LT outcomes in a VBHC perspective?		Carbone M
Measures of early postoperative course	Which are the best metrics to describe the quality of early postoperative course?		Polak WG
Measures of late postoperative course	Which are the best metrics to describe the quality of late post-transplant course?		Polak WG
Metrics of the whole transplant journey for outcomes comparison	Which is the best single measure to evaluate the whole LT process from the VBHC perspective?	If estimates of gain in life years or reduction in years lost are not available/calculable, what is the best measure to describe the transplant process from a VBHC perspective?	Cillo U, Carbone M

Expert panel of the Consensus Statement *Outcome measures in liver transplantation according to Value-Based Health Care* included: Carbone M, Neuberger JM, Rowe I, Polak WG, Forsberg A, Fondevila C, Mantovani L, Nardi A, Colli A, Rockell K, Schick L, Cristoferi L, Strazzabosco M, Cillo U.

ESOT selected a panel of experts to use a VBHC approach to develop a proposal for a core set of metrics reflecting the whole process of LT from candidate referral and listing to transplant and post-transplant care.

Only adult, elective liver, first transplantation from deceased donors was considered. Liver transplantation of children, for fulminant hepatic failure and transplants using living donors were not considered because their complexity requires specific sets of measures.

Due to the nature and novelty of the topics treated, and substantial lack of published evidence, the analysis was not developed using the PICO process [21]. Instead, we undertook a systematic review of the published metrics in LT, to select relevant evidence and to draft “good clinical practice recommendations” according to the GRADE definition. A literature search was done by expert staff from the Centre for Evidence in Transplantation (CET) who have expertise in conducting systematic reviews and these reviews were subsequently integrated, when needed, by the working group experts.

The search strategy used was as follows:

1. value based care.mp.
2. value based medicine.mp.
3. value of life/
4. cost-benefit analysis/ec, mt, st
5. Quality-Adjusted Life Years/
6. (quality adjusted adj2 life years).ti,ab.
7. survival benefit.ti,ab.
8. Intention to Treat Analysis/ec, mt, st
9. (life expectancy adj2 gain).ti,ab.
10. QALY.ti,ab.
11. quality metric.ti,ab.
12. or/1–11
13. Models, Statistical/
14. model$.ti,ab.
15. Benchmarking/
16. decision analysis.ti,ab.
17. or/13–16
18. liver transplantation/
19. liver transplant$.ti,ab.
20. 18 or 19
21. 12 and 17 and 20
22. remove duplicates from 21

The search strategy was focused on: systematic reviews, randomised controlled trials, registry analyses, observational prospective and retrospective studies, diagnostic studies, guidelines and official reports from UNOS, and other national transplant agencies, qualitative studies.

Exclusion criteria included: any language other than English; studies published before 1990.

The Transplant Library (TL), Medline and Embase were searched on 29 June 2022. The TL includes all randomised controlled trials and systematic reviews in the field of solid organ transplantation, whether published as full text or in abstract form, sourced mainly from MEDLINE/PubMed and hand-searches of congress proceedings.

After discussion in several virtual meetings, the panel formulated eight questions, that were presented during the ESOT conference held in Prague, Czech Republic, 13–15 November 2022. The response to these questions, presented as statements, were further discussed, modified until the best possible agreement was reached, and then voted by a selected jury. The questions and the final statements are reported and discussed in this manuscript.

## Results

Statements will be presented prioritizing those metrics describing the whole transplant process (Table 2). The subsequent statements refer to metrics referring to the various transplant phases following the trajectory of the patient along the liver transplant journey, starting from the time of listing for transplant. In this work we will not discuss the problem of the appropriate indications and timing for referral for LT evaluation.

**TABLE 2:** Topics and statements with rates of panel agreement.

Topic	Statements		Agreement (%)
Metrics referring to liver transplant as a whole			
Metrics of the whole transplant journey for outcomes comparison	Statement 1.1 From the *patient perspective*, intention to treat (i.e., from the patient listing) *gain in life years—*preferably, *quality of life-adjusted—*enables to describe the transplant process as a whole, since it reflects all the phases of LT from patient listing to the long-term postoperative course and expresses the benefit on alternatives		100
	Statement 1.2 From the point of view of *other transplant stakeholders*, an analysis from the point of transplant may be required. In this case, *gain in quality-adjusted life years*, should be the adopted metric. *Life-years lost* compared with healthy age- and sex-matched subjects provides further information on long term outcomes		
	Statement 2 In the absence of estimates of gain in life years or reduction in years lost, outcomes (for example, mortality or graft loss) should be calculated from the point of listing (i.e., ITT survival), as ITT takes into account multiple phases, i.e., patient selection, waiting list dynamics, allocation and acceptance of organs, and transplant outcome		
Timeframe for outcomes comparison	Statement 3 From the patient’s perspective, when assessing the whole transplant journey, the best timeframe for outcomes comparison should be at least 5 years and ideally 10 years, to balance urgency and utility		82
Single transplant phase metrics			
Quality of life after LT	Statement 4 Clinicians and researchers should be encouraged to use a generic instrument to measure quality of life in patients with chronic liver disease and after liver transplantation. Among the generic instruments, the EQ-5D is recommended, since it can be applied to all phases of transplantation, it is readily and freely available and validated across different countries		100
Patient reported outcome and experience measures	Statement 5 A core of validated PROMs, tailored to the sequential phases of the transplant journey, should be co-produced with public and patient involvement. This core of PROMs should be relevant to both clinical trials and routine healthcare		100
Waiting list management	Statement 6.1 In discussing the principles of waiting list management in LT, it is fundamental to underscore the importance of inclusion, diversity, equity		91
	Statement 6.2 Patient-reported experiences including managing expectations, providing appropriate education, responding to patient needs, efficient care, and maintaining communication should be assessed while patients are waiting for liver transplantation		100
	Statement 6.3 Wait list events including mortality, removal for deterioration, removal for improvement, temporary removal and removal for transplant should be recorded. These events should be subsequently processed in a competing risk analysis taking into consideration the centre case mix adjusted at the moment of listing and measuring the ability of the centre to accept higher risk patients.		91
Measures of early postoperative course	Statement 7 The metrics suggested to describe the quality of early postoperative course are early mortality and morbidity		100
	- 90 days mortality		
	- 90 days re-transplantation rate		
	- Length of the stay in the ICU		
	- Length of the stay in the hospital		
	- Readmissions rate		
	- Surgical/radiological re-interventions rate		
	- Clavien Dindo and the comprehensive complication index (CCI)		
	- Vascular and biliary complications rate		
	- Infectious complications rate		
	- 1 year mortality		
Measures of late postoperative course	Statement 8 The metrics suggested to describe the quality of late postoperative course are morbidity and 5 and 10 years survival ITT. When the analysis is extended to the whole transplant journey, an intention to treat approach is mandatory		100

### Metrics Referring to the Transplant as a Whole

Question 1 Which is the best single measure to evaluate the whole LT process, from the VBHC perspective?

#### Statement 1.1

From the *patient perspective*, intention to treat (i.e., from the time of patient listing), *gain in life years (preferably quality of life adjusted),* best describes the transplant process as a whole, since it reflects all the phases of LT from patient listing to the long-term postoperative course.

(consensus: 100%)

#### Statement 1.2

From the point of view of *other transplant stakeholders*, an analysis from the point of transplant may be required. If this case, *gain in quality-adjusted life years*, should be the adopted metric. *Life-years lost* compared with healthy age- and sex-matched subjects provides further information on long term outcomes.

(consensus: 100%)

Gain in life-years from the time of transplant, estimated from candidate and graft data, might provide information about the quality-adjusted extra years of life that a given transplant procedure could be expected to provide for a given patient [22, 23]. This metric can also be useful for designing an effective organ allocation system, where offers are prioritized to candidates deemed to have a greater transplant benefit [23]. An additional advantage would be to evaluate candidate-specific treatment options, i.e., using the characteristics of a specific candidate for a range of several possible donor grafts such as grafts from ECD, non-ECD or living donors). This information, along with candidate health status and the likelihood of receiving various types of graft, could be used to make informed decisions about whether to rule out offers of certain types of donor grafts for a specific candidate.

Unmet needs: The development of models estimating the gain in life years and life-years lost would require prospective intent-to-treat studies focused on the comparison between transplant with sex- and age-matched individuals without disease. Studies on cost-effectiveness, and validation between centers and countries are also required. Whether one or two measures are appropriate to evaluate LT process as a whole, rather than a set of metrics that reflect the several layers of complexity of LT, should also be experimentally explored, as the determinant of health and HRQoL in the pre-transplant and post-transplant settings are different.

Question 2 If estimates of gain in life years or reduction in years lost are not available/calculable, what is the best measure to describe the transplant process from a VBHC perspective?

#### Statement 2

In the absence of estimates of gain in life years or reduction in years lost, outcomes (for example, mortality or graft loss) should be calculated from the point of listing (i.e., ITT survival), as ITT takes into account multiple phases, such as patient selection, waiting list dynamics, allocation and acceptance of organs, and transplant outcome. (See also Statement 4.)

(consensus: 100%)

From a patient perspective, mortality matters whether it happens before or after transplantation. Survival from the point at which a patient is listed for transplant, is important for clinicians, patients and regulators, although robust evidence of its value as compared with survival from transplant is still limited [24]. Emphasis on outcomes from the time of transplantation rather than from the time of listing means ignoring patients who are removed because of deterioration or death on the waiting list.

Paradoxically, transplant survival would be better if an offer of a graft is declined for an ill recipient and the death occurs on the waiting list rather than after transplantation. Furthermore, focusing outcomes from the date of listing rather than that of transplantation means shifting the focus from the procedure to the patient.

Analysis of mortality from listing can be undertaken considering LT as a time dependent therapy. However, this approach has some important drawbacks. For patients, the significance of time on the list is substantially different from time after LT: death on the list is “in competition” with LT while death after LT is not, risk factors could be different and the impact of donor characteristics on patient and graft outcomes can be assessed only for transplanted patients.

Alternatively, Time from listing to LT and Time from LT to death could be analysed separately and then results combined. In the former analysis death on the wait list, removal for deterioration (or, rarely, improvement) and LT should be analysed as competing events, patients who are still actively waiting for a transplant are censored at that time. Any periods of suspension are not included in the waiting time [25].

Thus, for transplanted patients, analysis will focus on time from LT to death or re-LT, and risk factors, including donor characteristics, can be evaluated in the classical framework of survival analysis.

Unmet needs: When analysis of time from listing to LT and time from LT to death is undertaken separately, results in terms of survival probability should be combined. Probability rules can be applied but the most appropriate approach remains an open issue.

Not all patients will be listed at a similar time with respect to their risk of death and death, whether before or after transplantation, may be due to factors unrelated to the disease or its treatment. The impact of these issues should also be considered; corrections can be made using multivariate analysis and competing risks.

Question 3 What is the most appropriate timeframe to describe LT outcomes, from a VBHC perspective?

#### Statement 3

From the patient’s perspective, the best timeframe for outcomes comparison should be at least 5 years and ideally 10 years from the transplant to balance urgency and utility (Consensus: 82%).

While there is little doubt that, from the patient perspective, quality-adjusted long-term survival is the most relevant measure, it is important to keep in mind that, setting the time frame at 1, 3, 5 or 10 years will evaluate different aspects of the procedure and will be impacted by different risk factors. Donor factors, for example, are less important at 10 years than at 1 year. Furthermore, different healthcare professionals are often responsible for patient care at different time points of the transplant journey, thus making collection of consistent data a challenge.

Because 1 year survival exceeds 90% for most transplant indications, 1 year survival has a poor discrimination for center performance and has become more an expectation than a metric of performance. Also, a system focused on short-term outcomes (e.g., within 3 years) may lead centers to avoid higher risk recipients, a situation that undervalues the survival benefit of transplant. A system focused on long-term outcomes may incentivize centers to follow patients for longer periods. Managing the side effects of immunosuppression is key to continued patient health, with potential long-term sequelae of immunosuppression including an increased risk for malignancy, cardiovascular disease, and renal failure.

Moreover, long term outcomes will account for what matters most to patients; and these outcomes include physical function and social adaptation, return to work, mental wellbeing, and overall life satisfaction [26]. Most younger recipients will want to know life expectancy at 20 or 30 years if, for example, planning a family. A counterargument for the use of long-term follow-up is that this may be a poor metric for comparing outcomes of patients at different transplant centers as patients may choose (in countries where this is possible) not to be followed at the center where they underwent LT, and center should not be held accountable for outcomes of patients for whom they are no longer providing primary care. Furthermore, developments in the care of the transplant recipients means that extrapolation of patients grafted 20 years earlier may not be appropriate to patients about to undergo transplantation.

In summary, to provide a more comprehensive vision of the whole transplant procedure, the panel called for an extension of the outcome metrics to 5 and 10 years, but without discarding the outcome measurements currently collected at 1 and 3 years.

Unmet needs: Understanding which timeframe matters more to patients, linked to physical function, social adaptation including return to work, mental wellbeing, and overall life satisfaction should be further explored.

### Single Transplant Phase Metrics

Question 4 Which is the best tool to measure health-related quality of life, when assessing the benefit of liver transplantation?

#### Statement 4

Clinicians and researchers should be encouraged to use a generic and validated instrument to measure quality of life in patients with chronic liver disease and after liver transplantation. Among the generic instruments, the EQ-5D is recommended, since it can be applied to all phases of transplantation, it is readily and freely available and validated across different countries.

(consensus: 100%)

There is often a mismatch between the clinician’s assessment of the patient’s quality of life and the patient’s own assessment. Health-related quality of life is usually assessed using patient questionnaires or instruments [27–34]. These questionnaires should be relevant and acceptable to both patients and the general population and should use simple language, understandable to the patient and be culturally relevant. Questionnaires should also be simple, use as few questions as practicable, and be easily completed in a relatively short period of time. Furthermore, they should be validated in the population under evaluation, and be able to detect changes in health.

The assessments should be started at the time a patient reaches a stage when transplantation becomes an option, and repeated during assessment and at listing, and at agreed dates while awaiting and after transplantation. The European Network for Health Technology Assessment recommended that a generic HRQoL instrument is always used in clinical trials to cover a wide range of possible future uses of the HRQoL data [35]. We propose the use of EQ-5D in this setting as this is applicable in all phases of transplantation and is explicitly linked with health utility for cost-effectiveness analyses although we recognize it is neither specific for patients with liver disease nor after transplantation [36, 37].

Unmet needs: Although there are few published data, clinical experience confirms that areas of concern and their relative importance vary considerably depending on the stage of the patient’s journey. For example, concerns over the risks of donated organs are much less relevant post-transplant. Furthermore, it is important to distinguish between how the patient experiences the care they receive and the interactions with the transplant center (see below) from those related directly to the medical aspects of the transplant (such as side-effects of immunosuppressive drugs). This should be taken into consideration when developing specific HRQoL instruments.

Question 5 What are the unmet needs in defining the critical PROMs and PREMs to be included in liver transplant “core” evaluation and clinical trial design?

#### Statement 5

A core of validated PROMs, tailored to the sequential phases of the transplant journey, should be co-produced with public and patient involvement. This core of PROMs should be relevant to both clinical trials and routine transplant follow-up.

(consensus: 100%)

There is consensus that the involvement of patients in co-producing research and in decision-making about their health and care is of critical importance [38, 39]. Patient-reported measures are the key element to patient-centered VBHC, and by focusing on measuring what matters to the patient, the value of both clinical care and research is increased. Broadly, there are two types of patient-reported measures: patient reported outcome measures (PROMs) and patient reported experience measures (PREMS). PROMs measure the individual’s perception of their own outcomes in the broadest sense whereas PREMs measure perceptions on services and the experiences of care [40–42].

The nature of LT, encompassing the patient’s journey from the time of registration on the waiting list to long-term post-transplant survival, highlights the need for longitudinal health related quality of life data [43]. However, most studies reported to date are most often cross-sectional analyses and pay little attention to the phase post-transplant [27, 44].

A general framework for the development of PROMs should include information from across the relevant health domains—physical, social, and mental. Furthermore, the core outcome set should include generic measures of health-related quality of life (such as EQ-5D), and disease specific tools or transplant specific tools (such as the Liver Disease QoL questionnaire), along with patient perspective measures that should include measures of symptom distress, illness perceptions and patient empowerment (such as the Brief Illness Perception Questionnaire and the Patient Empowerment Scale) [36]. Inclusion of PREMs such as being taken seriously and listened to, should also be considered to improve the patient experience of LT and compare experiences between different centers and jurisdictions [45].

Unmet needs: The most appropriate tools to measure outcomes and experiences in LT have not been fully defined. ESOT and other organizations, should encourage original research to co-produce patient reported outcome and experience measures applicable to all phases of the transplant journey to holistically assess aspects of care.

Question 6 In a setting with optimal potential candidate, referral and listing process, which is the best measure to evaluate the quality of waiting list management from a VBHC perspective?

#### Statement 6.1

It is of fundamental importance to underscore the importance of inclusion, diversity, equity in the access to the liver transplant waiting list.

(Consensus: 91%)

Several studies have shown there are important inequities of access to transplant, based on racial and socioeconomic disparities [46, 47]. Inequalities may be due to a number of factors and vary by jurisdiction. There is inequity of access at all stages of the journey. There is also the inequity around insurance, outcome and possibly allocation of organs. ESOT adheres to the principle of health equity, and therefore rejects any limitations driven by socio-economic and racial/ethnic disparities that impact on access to transplantation. UNOS has developed an Access to Transplant Score (ATS) that indicates the likelihood for a waitlist candidate to receive an organ and this integrates with the NIMHD (National Institute Minority and Health Disparity) framework [48].

Unmet needs: an index measuring the existence of disparities in the listing process should be developed at the European level. These should mainly focus on access to the waiting list, but there are also inequities in waiting time and chance of dying on the list. Therefore, these should be measured as well.

#### Statement 6.2

Patient-reported experiences including managing expectations, providing appropriate education, responding to patient needs, efficient care, and maintaining communication should be assessed while patients are waiting for liver transplantation.

(Consensus: 100%)

The VBHC model requires consideration of the patient’s perspective, the clinical outcomes, and the costs. Patients may spend considerable time on the waiting list involving great uncertainty, often after a lengthy and difficult candidacy evaluation [49]. Therefore, the quality of life and the patients’ experiences while on the list must be measured and managed and be an important component of the evaluation.

This aspect has not been studied systematically [50, 51]. However, in a recent qualitative study [39], five themes emerged as patient priorities while on the list:

1. *Managing expectations*: most patients feel overwhelmed and want a clear description of the path ahead and how to navigate the process and relate to their healthcare providers. Centres must be respectful of the time involved going through the listing process, which can be substantial.
2. *Providing information*: listed patients remarked that lack of adequate information is a major determinant of anxiety on the waiting list. Information should be person-centred, comprehensive, transparent, relevant, and current.
3. *Responding to patient needs*: patients value highly responsive providers who deliver timely, personalized care able to compensate for eventual inefficiencies of the system.
4. *Executing the plan of care efficiently*: avoid delays, respect the patient’s time and avoid further financial burden to the patient.
5. *Maintaining effective interdisciplinary communication and coordination of care.* Patients view coordination of care as an extremely sensitive and important issue.

Unmet needs: Patient-reported experience measures (PREMs) should be co-developed in a collaboration between patients and professionals to the pre-transplant period to enable evaluation and improvement of waiting list.

#### Statement 6.3

Waiting list events including mortality, permanent removal because of death, deterioration or improvement, temporary removal and removal because of transplant should be recorded both at the center and national levels using a common data base and dictionary. These events should be processed in a competing risk analysis taking into consideration the centre case mix adjusted at the time of listing and measure the ability of the centre to accept higher risk patients. These events should be analysed and published by an independent group with patient and clinical and other input.

(Consensus: 91%)

The OPTN Board of Directors has recently published a briefing paper on how to enhance performance monitoring systems [52]. Although there are variations according to the jurisdiction and allocation system, ideally, a centre should make publicly available each year the number of patients who are removed from the list (because of improvement, deterioration, death) and the number of transplants done each year, but this should not prevent listing all those in need of a transplant if they fulfil the nationally agreed criteria. From the VBHC point of view, a transplant centre should be evaluated by how efficiently and equitably it provides for the listed patients and fulfils the commitment stipulated at the time of listing. Germane to these concepts would be the adoption of an intention-to-treat analysis when evaluating the transplantation results, as proposed in several of the following statements.

Unmet needs: Development of informatics tools to easily record the above parameters is essential. In the absence of such tools, data collection and recoding become labor-intensive and impacts negatively on already overburdened transplant teams. It was strongly recommended that the data listed above be made public.

Question 7 Which are the best metrics to describe the quality of early postoperative course?

#### Statement 7

The metrics suggested to describe the quality of the specific fraction of early postoperative course are early mortality and morbidity.

(Consensus: 100%)

There are no single metrics available to describe the quality of early postoperative course after LT. Ninety-day survival is one of the most informative, but to better capture the early post-operative course, the panel suggested adding a few simple but comprehensive set of metrics that are easy to obtain. While some of these metrics are not directly related to the Value Based approach and are not related to the patients’ experience, they remain essential to monitor and troubleshoot the process:

- 90 days mortality
- 90 days re-transplantation rate
- Length of the stay in the ICU
- Length of the stay in the hospital
- Readmissions rate (within 6 months)
- Surgical/radiological re-interventions rate (within 6 months)
- Clavien Dindo and the comprehensive complication index, CCI (within 6 months)
- Vascular and biliary complications rate (within 6 months)
- Infectious complications rate (within 6 months)
- 1 year mortality

Unmet needs: Although these metrics are available in most liver transplant centers, there is a need to harmonize their definition and expected values across the jurisdictions. Furthermore, there is a need to develop metrics for measuring the patients’ satisfaction with care during the early post-transplant recovery.

Question 8 Which are the best metrics to describe the quality of late post-transplant course?

#### Statement 8

The metrics suggested to describe the quality of late postoperative course are morbidity and mortality at 5 and 10 years.

(Consensus: 100%)

There are no single metrics available describing the quality of the long-term course after LT. It is suggested to adopt a few simple but comprehensive set of metrics that are easy to obtain, objective, quantifiable, verifiable, and validated such as:

- 5 years risk adjusted patient survival probability from listing for adult elective first liver registrations.
- 10 years risk adjusted patient survival probability from listing for adult elective first liver transplantation.

In addition to survival, morbidities after LT impact significantly on the patient and can be captured and measured as:

- Rate of chronic ductopenic rejection
- Recurrence of initial disease (such as autoimmune, viral, alcohol, steatotic liver disease)
- Rate of chronic renal dysfunction
- Rate of *de novo* diseases (such as systemic hypertension and dyslipidemia)
- Rate of *de novo* T2DM (NODAT)
- Rate of cardiovascular events
- *De novo* malignancies

Unmet needs: There is a need to agree the definition of many of these morbidities (such as what constitutes a relevant cardiovascular event or what degree of chronic renal impairment should be recorded). Informatics tools to easily record the above data are required. In the absence of these aids, data recording becomes too labor-intensive and impacts negatively on the already overburdened transplant teams.

## Discussion

Monitoring performance and reporting outcomes after liver transplantation is crucial for several reasons. First, it enables patients to make well-informed decisions about the outcomes, benefits and risks of transplantation. Second, it promotes an effective utilization of resources, including that of donated organs, and provides important feedback to the health authorities. Third, it helps clinicians monitor the process and promptly address issues. Fourth, a life that is gained should also be lived and factors of concern (such as severe symptom distress) that reduces HRQoL should be addressed. Moreover, transparent reporting is necessary to promote fairness and enhance transparency. As a result, multiple metrics have been implemented to promote performance and outcomes in liver transplantation [53].

However, the process of measuring and comparing outcomes after transplantation is intricate, and a single approach or metric cannot provide a comprehensive overview. When employed appropriately, these metrics are highly valuable in promoting the effective utilization of limited resources and facilitate the sharing of best practice. However, if used improperly, such measurements can lead to erroneous or misleading conclusions, foster risk-averse behavior, and hinder innovation and research [54].

Development of adequate metrics in liver transplantation can be daunting, given the variability of clinical situations, organs, jurisdictions, technologies, case mix and predictors. Additionally, different factors, such as characteristics of the donor, recipient, and surgical aspects, may have variable impacts on survival at different points in time. Differences in outcomes can be influenced, at least in part, by variations in case mix rather than variations between or within a specific transplant center. Risk adjustment models aim to account for these variations by incorporating relevant and validated risk-factors. This approach ensures that the risk profile of patients is appropriately considered when assessing outcomes and provides a more accurate evaluation of center performance [55–57].

Publishing transplantation outcomes is positive, but simplistic interpretation and utilization of data can be more detrimental than not publishing analyses, leading to risk-averse behavior, reduced transplant benefits, discouragement of research and a lack of innovation. Furthermore, when a metric become the objective, it stops being a useful metric [58].

In the past, performance monitoring of liver transplantation focused solely on post-transplant outcomes. However, there is now a growing trend towards analyzing outcomes starting from listing, which provides a more comprehensive understanding of the transplant process. This emerging approach is still in its developmental stages, with ongoing efforts to define the most clinically relevant methods of analyzing and presenting the data. It is important to note that analyses should also consider that patients may be listed at different times in relation to their risk of death, and that deaths, whether before or after transplantation, can be caused by factors unrelated to the disease or its treatment [59].

Recognizing these concerns, ESOT brought together an international group of experts, clinicians, researchers, and patient advocates from around the world to engage in rigorous discussions and critical analysis. The aim was to explore alternative outcome measures that provide a more holistic and patient-centered understanding of the transplantation process from a VBHC perspective.

VBHC conceptual approach is rapidly diffusing in most clinical disciplines since it aggregates the different phases of the therapeutic approach in a more comprehensive view of the full therapeutic process. The aim of VBHC is to measure ethical, societal and financial values according to what really matters to the patients. VBMH metrics are engineered to capture the perspective of the patient, and therefore are less granular than the indicators discussed before. We believe that an agreement on how to measure patient-centered value in liver transplantation is urgently needed also to subsequently perform fair benchmarking analysis.

The VBHC approach supports important changes in how patients, clinicians, commissioners, and researchers measure the quality of liver transplantation. These stakeholders have different needs.

Given the absence of published evidence concerning the effectiveness of implementing a Value Based approach, our approach was generated as consensus among experts. The panel formulated eight questions that lead to eight statements. The questions and statements have been further refined during the discussion at the ESOT meeting in Prague in 2022. These questions are formulated along the journey of a patient referred for liver transplant consideration. This is a first step, as VBMH mandates to develop PROMs, PREMs and costs to fully assess the value of the care.

Much work lies ahead, especially in the areas of cost studies and quality of life research. However, we hope that our effort will lay the foundation for implementing a VBHC approach in liver transplantation, addressing the critical need for a comprehensive framework in this field.

Considering that many of the patients have some difficulty understanding health information and navigating the healthcare system, health systems will have to address health literacy [60].

Finally, it should be highlighted that the costs associated for the development and implementation of such programs are not insignificant in terms of both human resources and healthcare funding; however, the benefit in quality of care provided to patients and the subsequent cost savings from prevention of complications, and readmissions, are posed to increase overall value.

## Data Availability

The original contributions presented in the study are included in the article/supplementary material, further inquiries can be directed to the corresponding author.

## Abbreviations

ATS, access to transplant score; CCI, comprehensive complication index; CET, Centre for Evidence in Transplantation; EASL, European Association for the Study of the Liver; ERCP, endoscopic retrograde cholangiopancreatography; ESOT, European Society of Organ Transplantation; ELITA, European Liver and Intestine Transplant Association; EKITA, European Kidney Transplant Association; EPITA, European Pancreas and Islet Transplant Association; ECTTA, European Cardio Thoracic Transplant Association; ETHAP, European Transplant Allied Healthcare Professionals; ICU, intensive care unit; MELD, model for end-stage liver disease; LT, liver transplantation; NIMHD, national institute minority and health disparity; NODAT, new-onset diabetes mellitus after transplantation; OPTN, Organ Procurement and Transplantation Network; PICO, Population/Intervention/Comparison/Outcome; PREMs, Patient-Reported Experience Measures; PROMs, Patient-reported outcome measures; PTLD, post-transplant lymphoproliferative disorder; QALY, quality-adjusted life year; RCT, randomized-controlled trial; UNOS, United Network for Organ Sharing; VBHC, value-based health care.
